# Supplementary material for: Founder transformants of cotton (Gossypium hirsutum L.) obtained through the introduction of DS-Red, Rec, Rep and CRISPR/Cas9 expressing constructs for developing base lines of recombinase mediated gene stacking
Source: PLoS One. 2022 Feb 3;17(2):e0263219. doi: 10.1371/journal.pone.0263219 (PMC8812945; doi:10.1371/journal.pone.0263219)

A

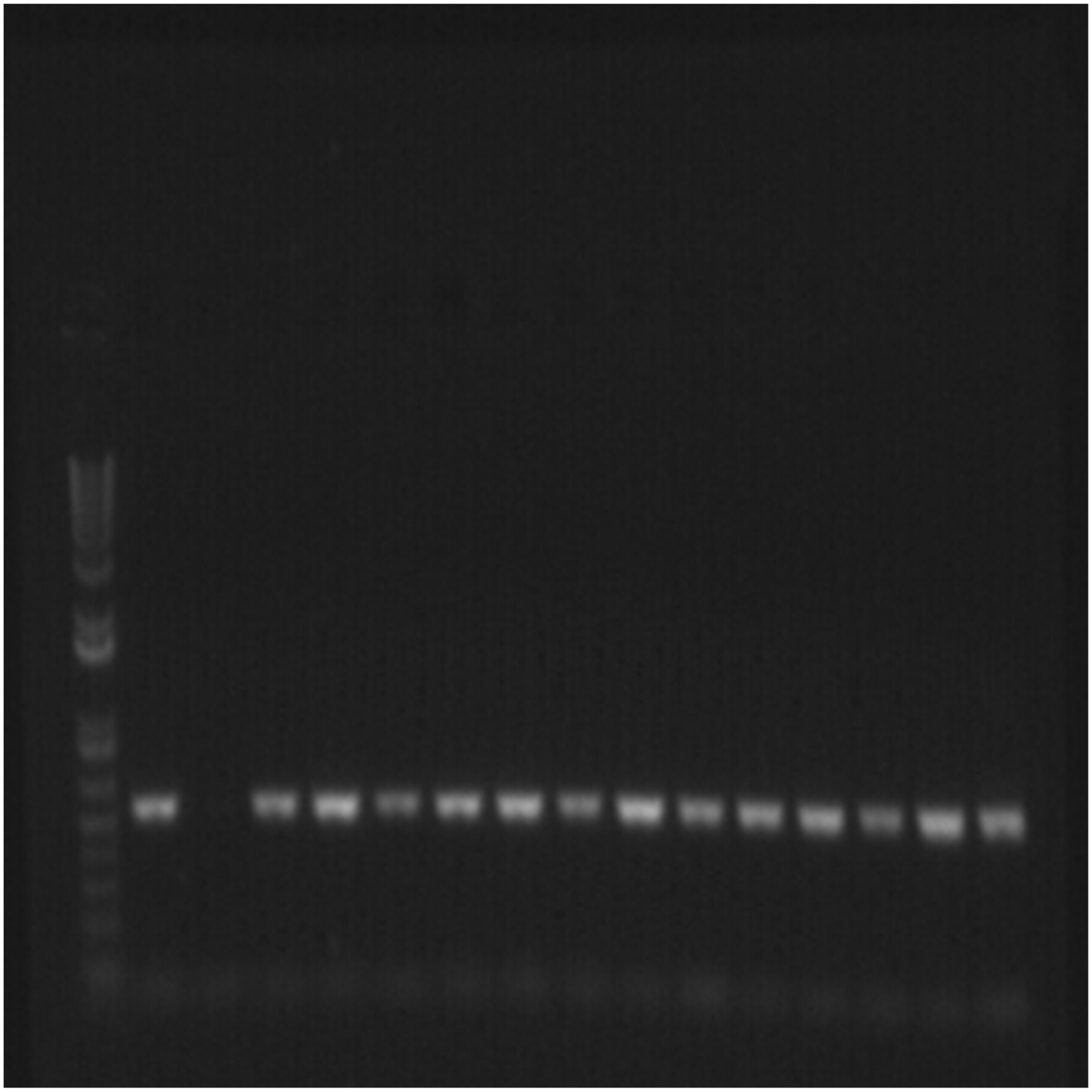

B

Nptll
pG-Rec
557 bp

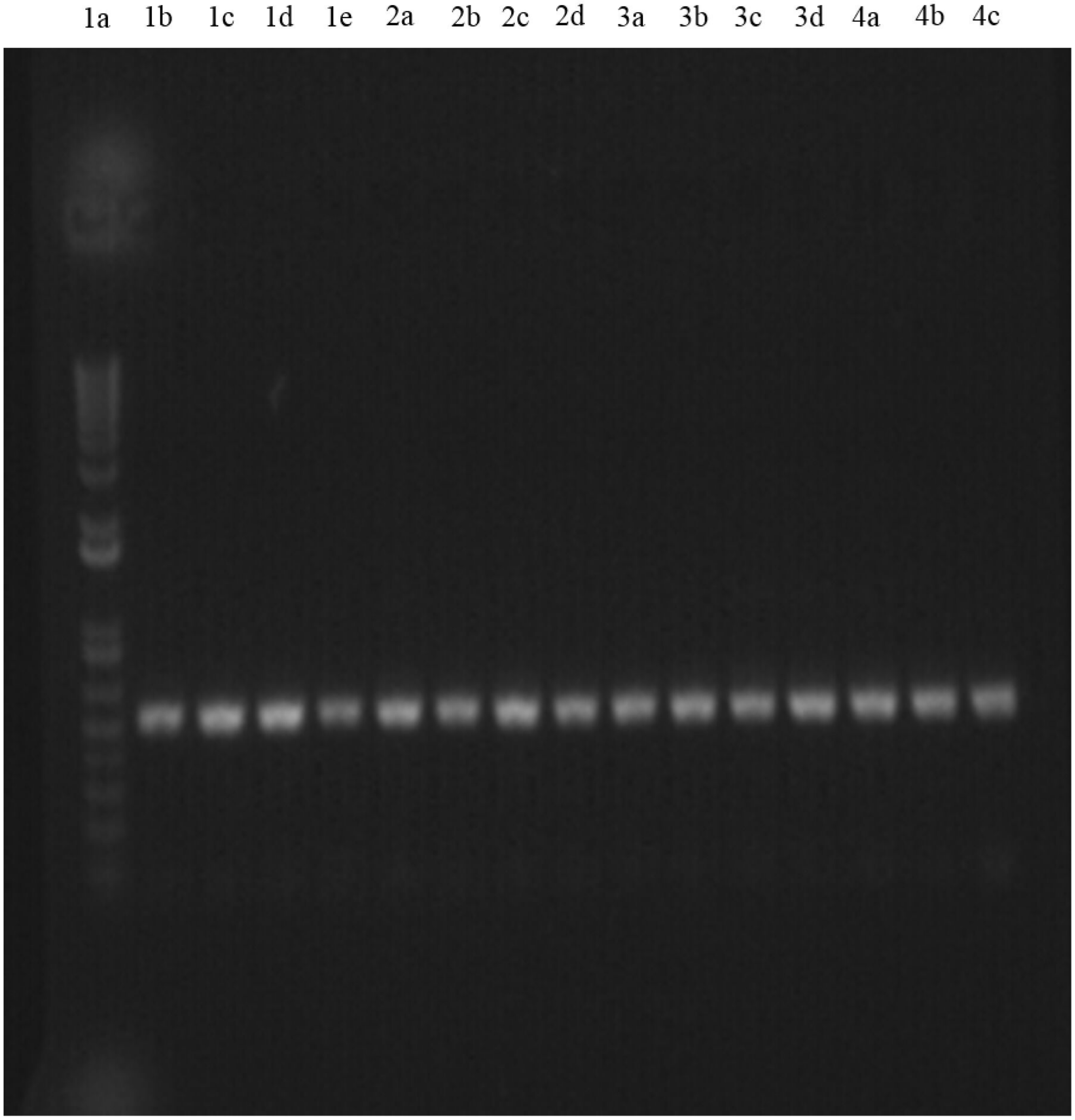

C

DS-Red
pKGW-RR
2489 bp

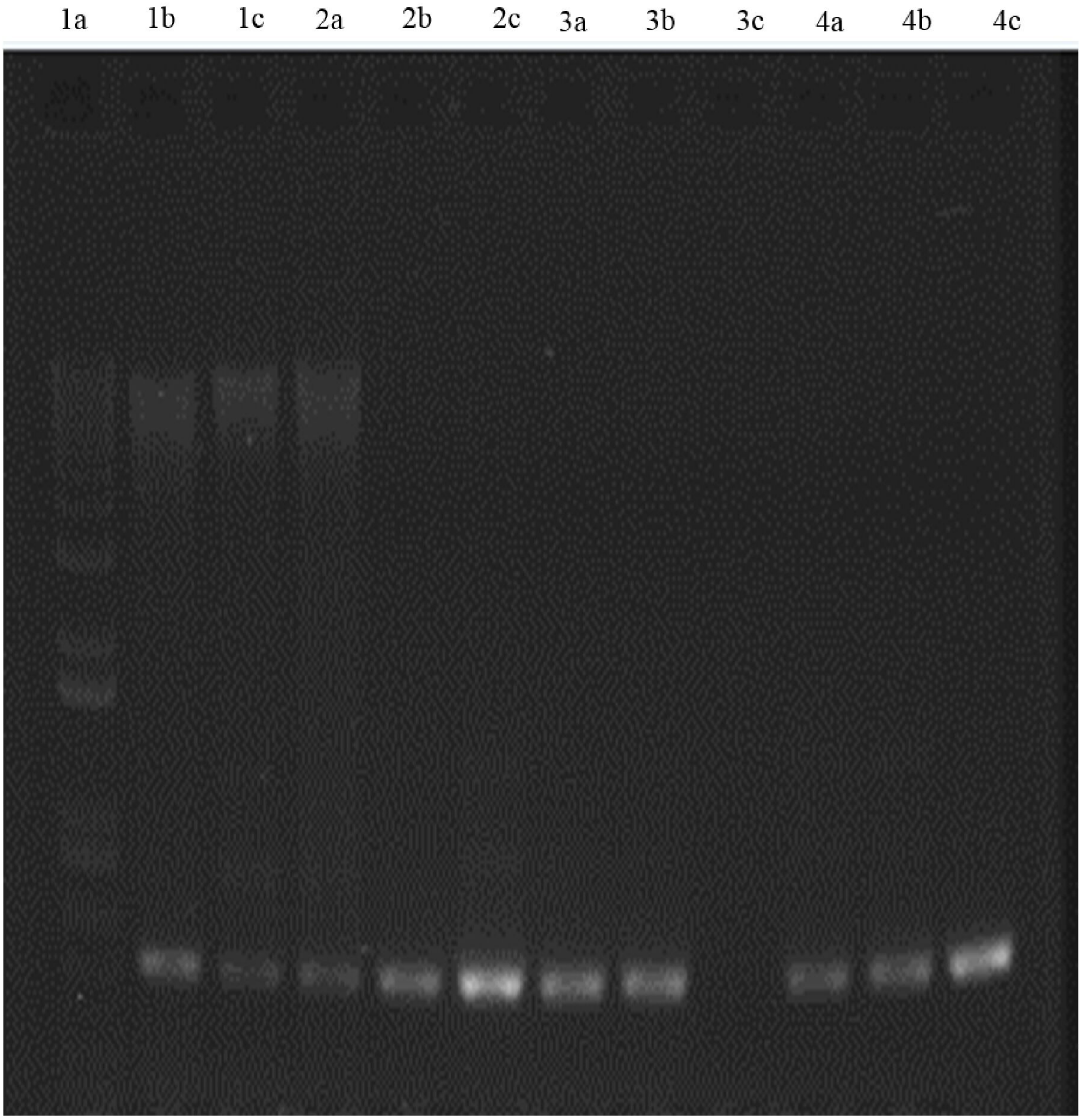

D

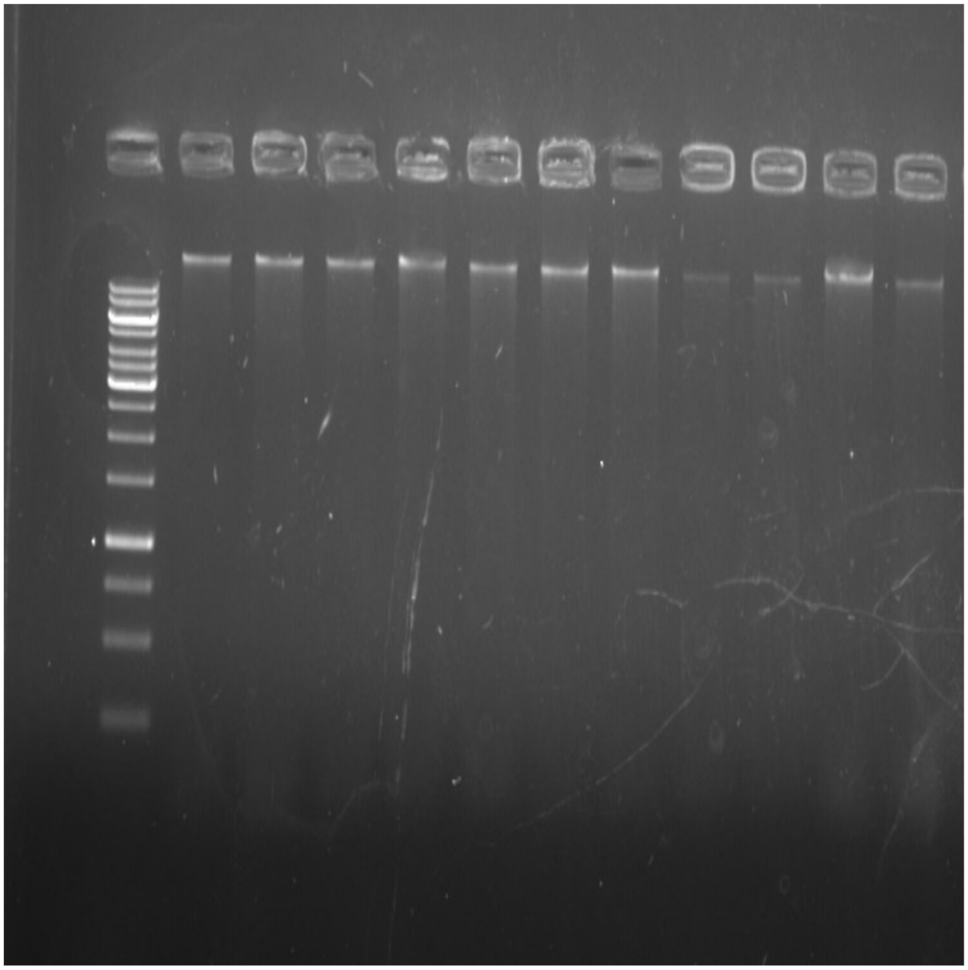

E

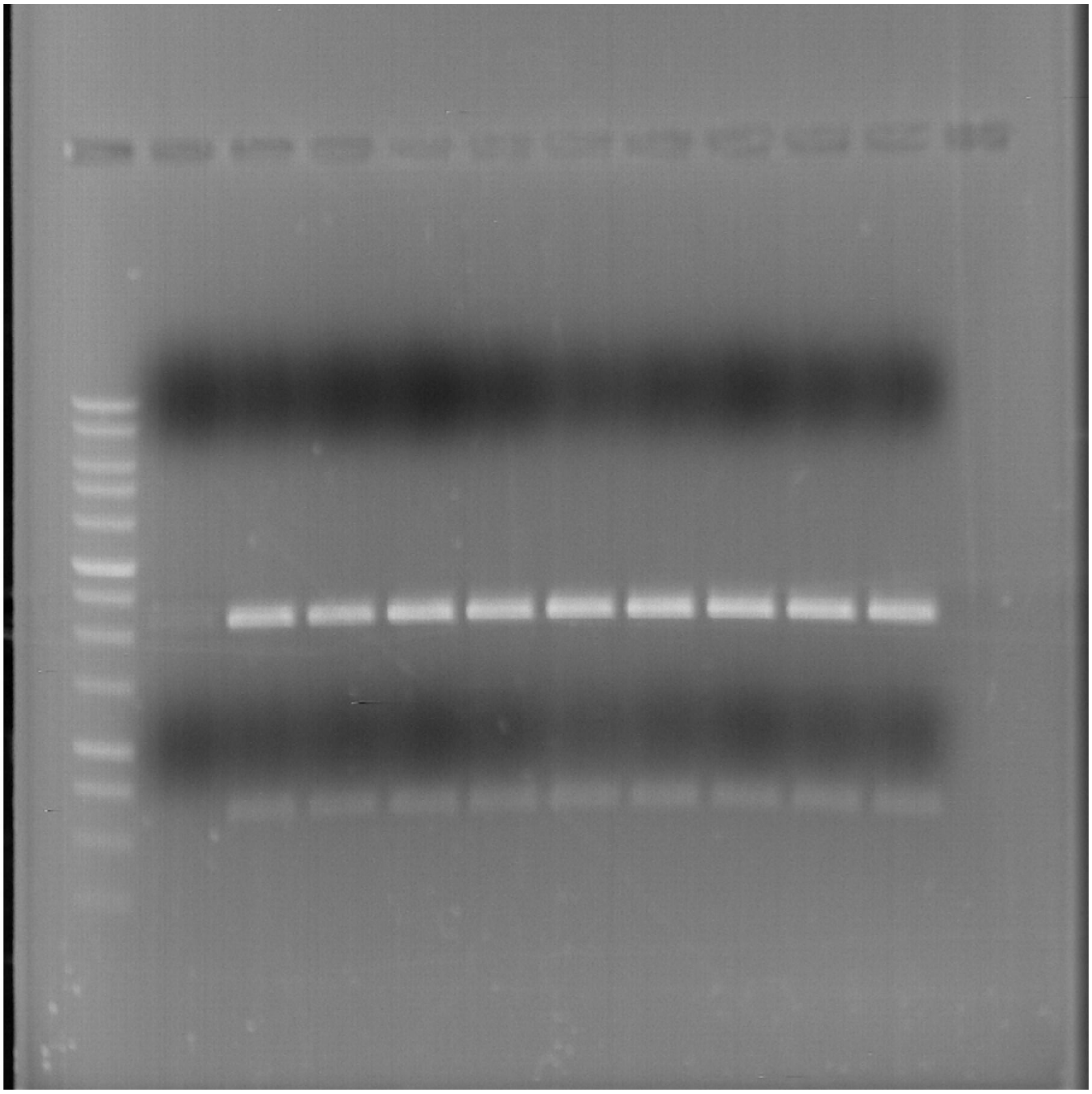

Cas9 gene
pHSE-401
4266 bp

F

Rec-cassette
pG-Rec
1493 bp

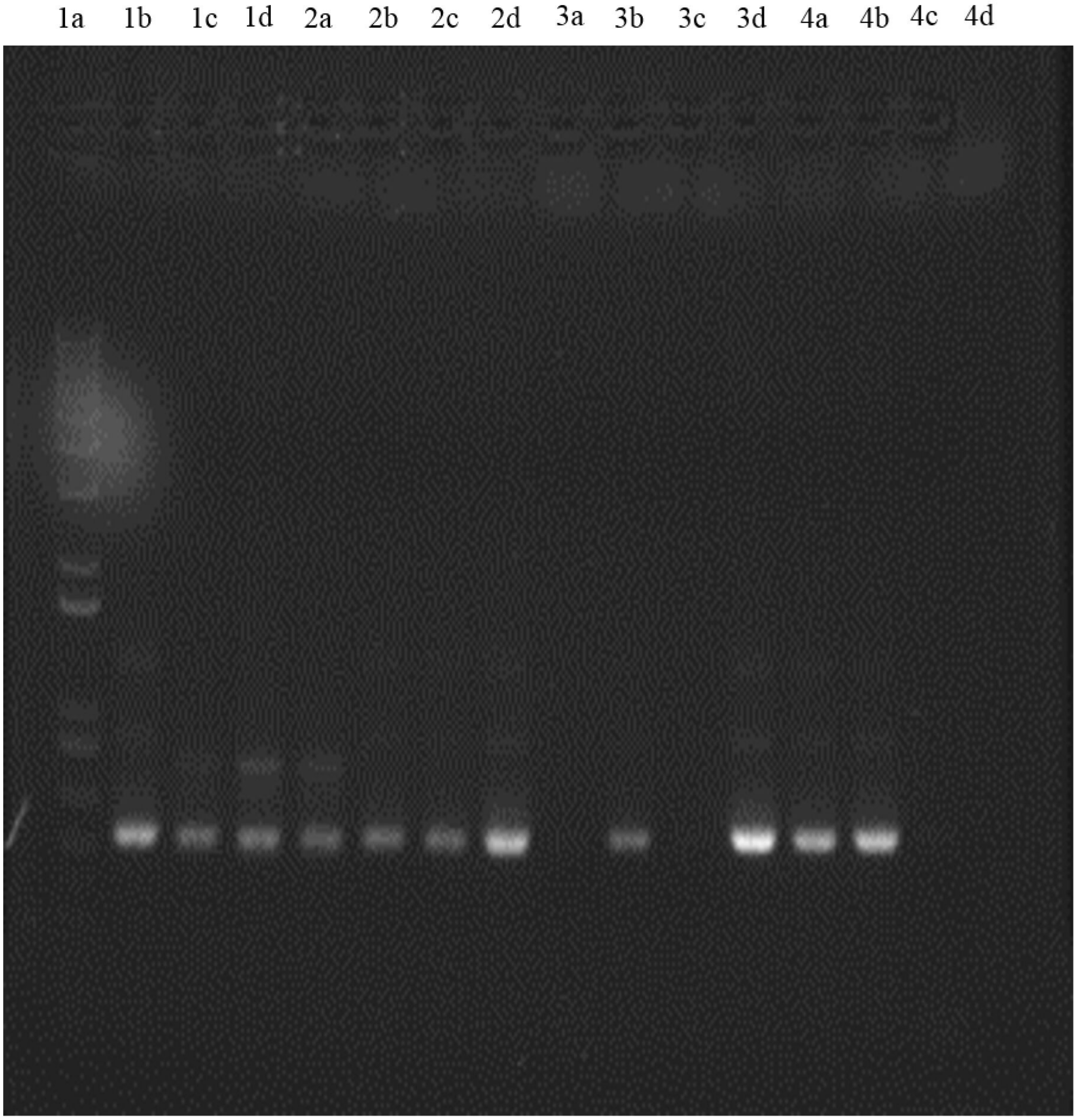

G

Rec-cassette

pCas-Rec

1449 bp

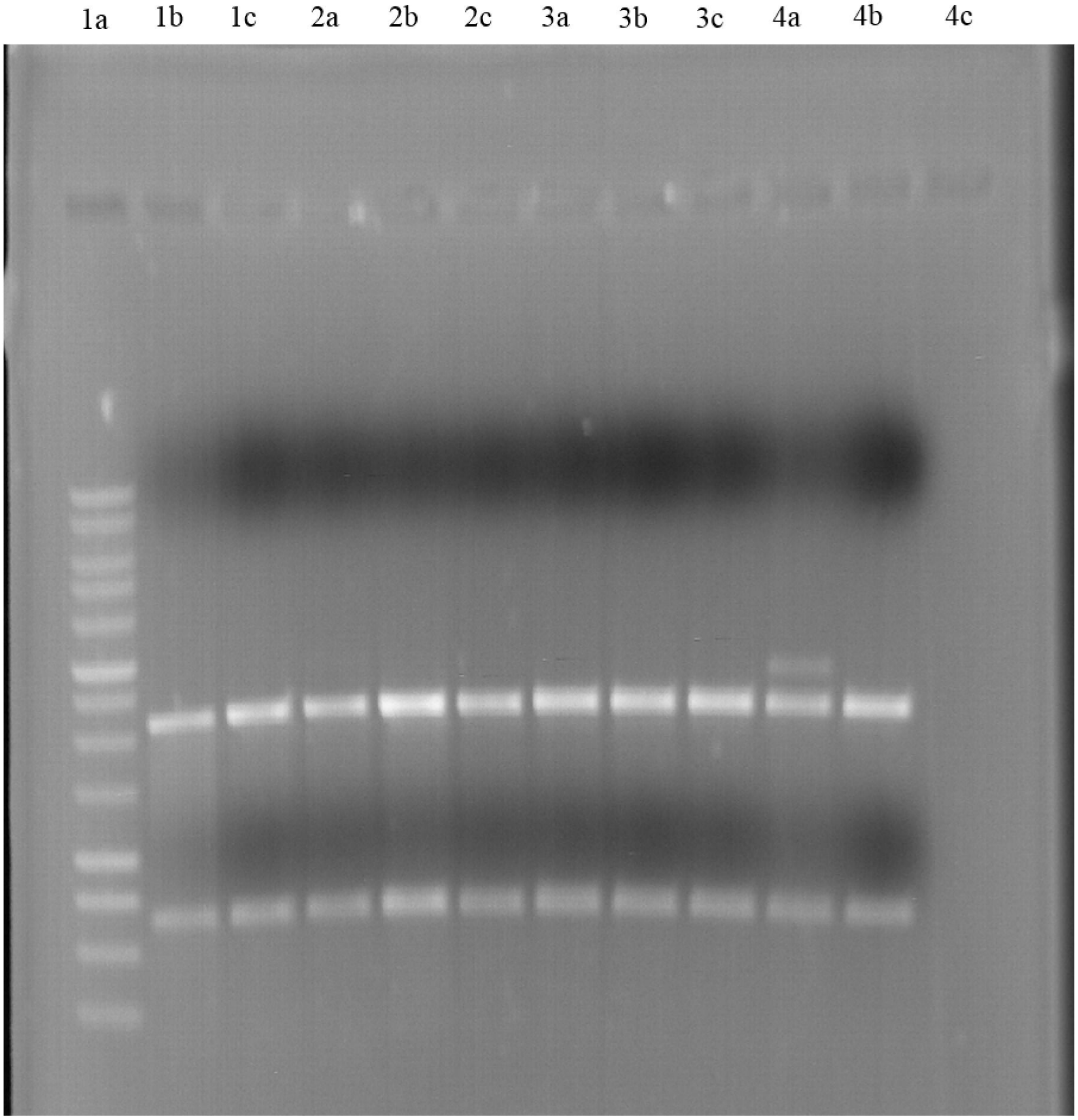

Supplement: S1 Raw images — (PDF) [file pone.0263219.s001.pdf]
