# Supplementary material for: Founder transformants of cotton (Gossypium hirsutum L.) obtained through the introduction of DS-Red, Rec, Rep and CRISPR/Cas9 expressing constructs for developing base lines of recombinase mediated gene stacking
Source: PLoS One. 2022 Feb 3;17(2):e0263219. doi: 10.1371/journal.pone.0263219 (PMC8812945; doi:10.1371/journal.pone.0263219)
Supplement: Attachment — Submitted filename: Response to reviewers letter.pdf [file pone.0263219.s003.pdf]

SABIN ASLAM

University of California, Davis, CA, USA 95618
Contact #: +1(530)760-6013 · +92-333-7675764
Email: sabaslam@ucdavis.edu · sabinaslam@gmail.com
LinkedIn Profile: https://www.linkedin.com/in/sabin-aslam-810b9932/

Dr. Joerg Heber
Editor-in-Chief *PLOS-ONE*

May 20th, 2021

Dear Dr. Heber,

I am contented to submit the revised research article entitled "Founder transformants of cotton (*Gossypium hirsutum* L.) obtained through the introduction of DS-Red, Rec, Rep and CRISPR/Cas9 expressing constructs for developing base lines of recombinase mediated gene stacking" for consideration of publication in PLOS ONE. The revisions have been done according to reviewers' suggestions as:

1. The manuscript format style was changed according to PLOS ONE style requirements.
2. All images were changed with unadjusted ones.

Reviewer 1:

1. All the comments from Reviewer # 1 are properly addressed.
2. The paper is now appropriately English edited and properly organized. The novelty of the articles implies its development of CRISPR/Cas9 based founder lines to be used as initiator lines of CRISPR based gene stacking projects. Using these founder lines could lessen 12-14 months of all the gene stacking research projects timeline. It also eliminates the time required for stable integration of Cas genes, each time.
3. Modified the materials and methods section according to reviewer suggestion.
4. Figure titles are modified.
5. These founder lines are useful in gene stacking projects and the research findings will be published in series with this being first step publication.
6. The manuscript represents technically sound piece of scientific research with rigorous experiments conducted with appropriate replications described in Table 2.
7. Statistical analysis has been included as Figure No. 7.
8. MS is now English edited by a professional English expert.
9. The method section has been modified with all the methods optimized by author herself.
10.

Reviewer 2:

1. Changed reference at line 46
2. Suggested research article discussed at lines 492-500
3. Explained at lines 170-175
4. Lines 197-198
5. Added at line 206 and then followed in whole materials and methods section
6. Organized according to suggestions
7. Lines 501-516
8. Lines 507-516
9. Lines 433-437
10. Suggested reference has been added
11. Figures are explained well and methods are cleared well.

Reviewer 3:

1. Two parentheses omitted
2. "Couldn't be" is replaced with "could not"
3. Page 4, line 70; for the eradication of disease completely: Added
4. Shortened in materials and methods section
5. All figure captions are modified to descriptive ones

We believe that this manuscript is appropriate for publication now by PLOS ONE because it is an important study to combat biological stresses of cotton. Our manuscript creates a paradigm for future studies of the CRISPR/Cas9 based targeting of plant diseases and numerous gene stacking projects.

This manuscript has not been published and is not under consideration for publication elsewhere. We have no conflicts of interest to disclose the manuscript. If you feel that the manuscript is appropriate for your journal, then please proceed to the publication process.

Thank you for your consideration!

Sincerely,

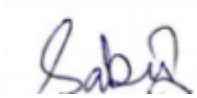

Sabin Aslam
