# Supplementary material for: Founder transformants of cotton (Gossypium hirsutum L.) obtained through the introduction of DS-Red, Rec, Rep and CRISPR/Cas9 expressing constructs for developing base lines of recombinase mediated gene stacking
Source: PLoS One. 2022 Feb 3;17(2):e0263219. doi: 10.1371/journal.pone.0263219 (PMC8812945; doi:10.1371/journal.pone.0263219)
Supplement: Attachment — Submitted filename: Response to reviewers letter30.08.21.pdf [file pone.0263219.s004.pdf]

SABIN ASLAM

University of California, Davis, CA, USA 95618
Contact #: +1(530)760-6013 · +92-333-7675764
Email: sabaslam@ucdavis.edu · sabinaslam@gmail.com
LinkedIn Profile: https://www.linkedin.com/in/sabin-aslam-810b9932/

Dr. Joerg Heber
Editor-in-Chief *PLOS-ONE*

August 30th, 2021

Dear Dr. Heber,
I am contented to submit the revised research article entitled "Founder transformants of cotton (*Gossypium hirsutum* L.) obtained through the introduction of DS-Red, Rec, Rep and CRISPR/Cas9 expressing constructs for developing base lines of recombinase mediated gene stacking" for consideration of publication in PLOS ONE. The revisions have been done according to reviewers' suggestions as:

1. The manuscript format style was changed according to PLOS ONE style requirements.
2. All images were changed with original uncropped and unadjusted ones. Any of the figures included in this manuscript aren't included in supporting information or in public data repository.

Reviewer 1:

1. MS is adjusted according to PLOS ONE style requirements.
2. Original uncropped and unedited images are provided.
3. All the comments from Reviewer # 1 are properly addressed.
4. The paper is now appropriately English edited and properly organized. The novelty of the articles implies its development of CRISPR/Cas9 based founder lines to be used as initiator lines of CRISPR based gene stacking projects. Using these founder lines could lessen 12-14 months of all the gene stacking research projects timeline. It also eliminates the time required for stable integration of Cas genes, each time.
5. Lines 19 and 20 are replaced with each other.
6. Line:41: reference added.
7. Line 47: 31.66mha area added.
8. Line 48: reference updated.
9. Line 49: new paragraph.
10. Line 50: "one such stressors" replaced with "a destructive biological agent."
11. Line 52: eighteenth replaced by twentieth.
12. Line 54: most devastated replaced with worsened.
13. Line 63: affects replaced by reduces.
14. Line 64: "by being a source of CLCuV but also diminishes plant growth by directly nourishing to reduce fiber quality traits" replaced with "plant growth and fiber quality."
15. Line 65: "primarily" added.
16. Line 68: "were not completely resistant" sentence alignment changed with "varieties varied in their degree of susceptibility."
17. Line 69: "It has been reported" removed.
18. Line 73: "wild" added.
19. Line 75: "at a single point" replaced with "specific site."
20. Line 86: removed "completely."
21. Line 80: added "a"
22. Line 83: "one" is replaced with "plant."
23. Line 84 "straight" replaced with "direct."
24. Line 84 "pile up" replaced with "stack."
25. Line 85 "precise location" replaced with "a precise location."
26. Line 86: "is called" replaced with "called."
27. Line 87: "is used" added before "to integrate."
28. Line 88 & 89: sequence of citations was arranged alphabetically.
29. Line 93: "to our system" removed.
30. Line 94: "to develop as" replaced with "for optimizing"
31. Line 95: "at that specific sit" replaced with "target location."
32. Line 96: "recombination" added.

33. Lines 103, 104 & 105: aligned alphabetically. "Apical meristems, cotyledons, embryogenic suspension cells, mature embryos, shoot apex, somatic and embryonic calli."
34. Line 105-110: citations arranged alphabetically. "Ali et al., 2004; Amudha et al., 2011; Bajwa et al., 2015; Chlan et al., 1995; Cousins et al., 1991; Finer and McMullen, 1999; Ikram-ul-Haq, 2004; Jin et al., 2005; Jing-Lin et al., 2008; Keshamma et al., 2008; Leelavathi et al., 2004; Mishra et al., 2003; Morre et al., 1998; Rajasekaran et al., 2000; Rech et al., 2008; Sohrab et al., 2014; Sunilkumar and Rathore, 2001; TianZi et al., 2010; Tohidfar et al., 2005; Wilkins et al., 2004 and Wu et al., 2005."
35. Line 116: "starting from developing the initial Cas9 plants: replaced with "developing the Cas9 plants from scratch."
36. Line: 118. "have" removed.
37. Line 120: "Development of cotton founder plants" replaced with "Next, we developed cotton founder plants."
38. Line 121: "was the second step of our study."
39. Line 121: "The" added.
40. Line 123: "The" added.
41. Line 127: "and" added.
42. Line 129: "That is" added.
43. Line 130: "and" added.
44. Line 135: "for checking the expression of desired gene" removed.
45. Line 136: "lessens" replaced with "mitigates."
46. Line 136: "caused by" replaced with "associated with."
47. Paragraph "four different plasmids" shifted from line 134 to 138.
48. Line 140: "courtesy of Dr. Douglas Cook, Feed the future innovation lab for climate resilient chickpea, UC-Davis" replaced with "Brendan et al., 2007."
49. Line 156: "The" added.
50. Line 161: "The" added.
51. Line 176-177: "Coker-312 seeds were germinated at 28°C in darkness for in sunshine mix soil moist with nutrient water" replaced with "The sunshine mix soil misted with nutrient water was used for Coker-312 seeds germination in dark at 28°C."
52. Line 177: "were watered continuously on" removed.
53. Line 180: "allowing them to start photosynthesis" replaced with "to initiate the process of photosynthesis."
54. Line 181: "from soil in dark."
55. Line 193: "*" removed.
56. Line 197: "to avoid."
57. Line 201: "proper" removed.
58. Line 208: "from" replaced with "of."
59. Line 223-224: parenthesis removed.
60. Line 225: "hypocotyls" relaced with "and"
61. Line 228 "on" replaced with "at"
62. Line 228: "(i.e 28°C and 16/8 hours)" replaced with "that is 28°C and 16/8 hours respectively."
63. Line 257: "On reaching 5-6 leaves stage" replaced with "When 5-6 leaves grew."
64. Line 257: "developing" replaced with "to develop."
65. Line 258: "for a brief period of time" replaced with "briefly."
66. Line 259: "the time of opening jars: was replaced with "frequency of opening jars"
67. Line 259 "health capacity" replaced with "health condition."
68. Line 264: "of the cotton plants" removed.
69. Line 279: (Table 1)" replaced with "in table 1."
70. Line 283: "plant" removed.
71. Line 284: "inveterate" was replaced with "inveterated"
72. Line 285: "DNA in bulk amount ~300-500µg/ sample" with "about 300-500µg DNA per sample.
73. Line 289: inserted "The."
74. Line 296: "gel was" was replaced with "and then"
75. Line 297: "wet" replaced with "immersed"
76. Line 299: "layed" replaced with "put"
77. Line 299: "was put at the top" and "approximately" were removed.
78. Line 300: "then" was added.
79. Line 306: "to" replaced with "at"

80. Line 308: “stringently” removed.
81. Line 309: “it” was replaced with “membrane”
82. Line 309: “membrane” was replaced with “and then”
83. Line 317: “is” replaced with “was”
84. Line 317: added “then”
85. Line 321-326 were moved to the beginning of results section.
86. Lines 329-351 were rewritten.
87. Line 355: “On observing callus appearance” was replaced with “as callus appeared”
88. Line 360: “the” added.
89. Lines 360-362: “pKGW-RR transformed somatic calli were visibly red. Embryos appeared as small, smooth whitish beads seen within coarse reddish callus in DS-Red transformed hypocotyls (while growing in dark).” Was replaced with “The pKGW-RR transformed somatic calli were visibly red. The embryos within coarse reddish callus in DS-Red transformed hypocotyls (being grown in dark) appeared as small and smooth whitish beads.”
90. Line 377: “i.e” replaced with “that is”
91. Line 379: “among brownish coarse callus” was removed.
92. Line 380: “turned green” was replaced with “helped them turn green”
93. Lines 381-382: “in 16/8 hours photoperiod for proper growth and development of embryos and for inducing them to start photosynthesis” replaced with “for a photoperiod of 16/8 hours to induce embryos’ growth, development and photosynthesis”.
94. Lines 382-383: “Embryos started elongating in size and developed green leaflets” replaced with “Embryos elongated, changed the shape from globular to torpedo and developed green leaflets”.
95. Lines 383-386: “When embryos reached to 10-12cm in length and had developed the first two leaflets, they were shifted to a CRIM having no growth regulators, hormones or amino acids, but added with respective selection agent” replaced with “When embryos developed the first two leaflets and attained the length of 10-12cm, they were observed on CRIM with no growth regulators, hormones or amino acids, but with added respective selection agent”
96. Line 386: “embryos” replaced with “they”
97. Line 386: “while keeping” replaced with “when kept”
98. Line 388: “controlling” replaced with “limiting”
99. Line 390: “turned to” replaced with “became”
100. Line 401: “to differentiate the NptII gene in bacteria or as T-DNA integrated into plant genome” replaced with “to differentiate the inherent gene of bacteria or as the whole T-DNA integrated into plant genome”
101. Line 405: “arises” replaced with “arose”
102. Lines 427-428: “Healthy calli separated from hypocotyls when selected on kanamycin plates appeared as red” was replaced with “Healthy calli separated from hypocotyls appeared as red when selected on kanamycin plates”.
103. Line 428: “was” removed
104. Line 429: “showed” replaced with “show”
105. Line 430: “might be” replaced with “it could be”
106. Line 435: “and transformation percentage per batch” was added.
107. Line 438-442: figure 7 and figure caption added.
108. Line 445: “carrying” replaced with “carried”
109. Line 456: “GOI translocate” replaced with “integrated cassette translocated”
110. Line 457: “integrated” replaced with “incorporated”.
111. Line 462: “substantiated” replaced with “was found”
112. Line 462: “showing” replaced with “identifying”
113. Line 463: “successfully” removed
114. Line 465: “ease the development of” replaced with “helped develop”
115. Line 468: “the” added
116. Line 468: “as an initial vector” replaced with initially.
117. Line 475: “integration” replaced with “addition”
118. Line 476: “with a subsequent process” replaced with “and”
119. Line 477: “an” removed.
120. Line 479: “provided, founder plants for initiating” replaced with “initiated”
121. Line 483: “to indicate the validity of this construct against virus” replaced with “to test its efficacy”

122. Line 492: “conclusively” replaced with “to conclude.”
123. Lines 495-496: “cotton bolls survival” replaced with “survival of cotton bolls”
124. Line 498: “caused by” replaced with “associated with”
125. Line 499: “workable” replaced with feasible”
126. Line 502: “sitting inside the genome of cotton” replaced with “residing in cotton’s genome”
127. Line 504: ‘in design strategy” removed
128. Line 507-508: “Some of the widespread CRISPR applications of cotton genome editing includes” added.
129. Line 510: “are widespread CRISPR application of cotton genome editing” removed.
130. Line 515: “of cotton” removed.
131. Modified the materials and methods section according to reviewer suggestion.
132. Figure titles are modified as suggested by reviewer 1.
133. These founder lines are useful in gene stacking projects and the research findings will be published in series with this being first step publication.
134. The manuscript represents technically sound piece of scientific research with rigorous experiments conducted with appropriate replications described in Table 2.
135. Statistical analysis has been included as Figure No. 7.
136. MS is now English edited by a professional English expert.
137. The method section has been modified with all the methods optimized by author herself.
138. Result section is aligned at its best.

Reviewer 2:

1. Changed reference at line 51
2. Suggested research article discussed at lines 504-512
3. Explained at lines 176-181
4. Lines 204-206
5. Added at line 212 and then followed in whole materials and methods section
6. Organized according to suggestions
7. Summarized on lines 513-528
8. Discussed in lines 519-528
9. Lines 443-448
10. Suggested reference has been added
11. Figures are explained well and methods section is cleared well.

Reviewer 3:

1. Two parentheses omitted
2. “Couldn’t be” is replaced with “could not”
3. Page 4, line 70; for the eradication of disease completely: Added
4. Shortened southern blot analysis in materials and methods section
5. All figure captions are modified to descriptive ones
6. All original unedited pictures have been added

We believe that this manuscript is appropriate for publication now by PLOS ONE because it is an important study to combat biological stresses of cotton. Our manuscript creates a paradigm for future studies of the CRISPR/Cas9 based targeting of plant diseases and numerous gene stacking projects.

This manuscript has not been published and is not under consideration for publication elsewhere. We have no conflicts of interest to disclose the manuscript. If you feel that the manuscript is appropriate for your journal, then please proceed to the publication process.
Thank you for your consideration!
Sincerely,

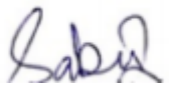

Sabin Aslam
